# Investigating the genetics of *Bti* resistance using mRNA tag sequencing: application on laboratory strains and natural populations of the dengue vector *Aedes aegypti*

**DOI:** 10.1111/eva.12082

**Published:** 2013-08-31

**Authors:** Margot Paris, Sebastien Marcombe, Eric Coissac, Vincent Corbel, Jean-Philippe David, Laurence Després

**Affiliations:** 1 Laboratoire d'Ecologie Alpine (LECA), UMR 5553 CNRS-Université de GrenobleGrenoble, France; 2 Plant Ecological Genetics, Institute of Integrative Biology, ETHZurich, Switzerland; 3 UMR MIVEGEC (UM1-CNRS 5290-IRD 224), Institut de Recherche pour le Développement (IRD)Montpellier, France

**Keywords:** *Aedes aegypti*, *Bacillus thurigiensis* var. *israelensis*, genome scan, insecticide resistance, next-generation sequencing, RNAseq

## Abstract

Mosquito control is often the main method used to reduce mosquito-transmitted diseases. In order to investigate the genetic basis of resistance to the bio-insecticide *Bacillus thuringiensis* subsp. *israelensis* (*Bti*), we used information on polymorphism obtained from cDNA tag sequences from pooled larvae of laboratory *Bti*-resistant and susceptible *Aedes aegypti* mosquito strains to identify and analyse 1520 single nucleotide polymorphisms (SNPs). Of the 372 SNPs tested, 99.2% were validated using DNA Illumina GoldenGate® array, with a strong correlation between the allelic frequencies inferred from the pooled and individual data (*r* = 0.85). A total of 11 genomic regions and five candidate genes were detected using a genome scan approach. One of these candidate genes showed significant departures from neutrality in the resistant strain at sequence level. Six natural populations from Martinique Island were sequenced for the 372 tested SNPs with a high transferability (87%), and association mapping analyses detected 14 loci associated with *Bti* resistance, including one located in a putative receptor for Cry11 toxins. Three of these loci were also significantly differentiated between the laboratory strains, suggesting that most of the genes associated with resistance might differ between the two environments. It also suggests that common selected regions might harbour key genes for *Bti* resistance.

## Introduction

Mosquito control is an important component of vectorborne disease control, as mosquitoes transmit serious diseases to humans such as malaria, filariasis and dengue fever. After the rapid emergence of mosquito resistance to all classes of chemical insecticides (Hemingway et al. 2004; Ranson et al. 2010), the bacterio-insecticide *Bacillus thuringiensis* subsp. *israelensis* (*Bti*) provided as a safe and efficient alternative across the world (Lacey and Siegel 2000) and since 2007 has been used exclusively by most European countries. *Bti* has also been used since 2007 to control the dengue vector *Aedes aegypti* in tropical regions such as the French West Indies. During sporulation, *Bti* bacteria produce a solid parasporal crystal composed of insecticidal toxins (Cry and Cyt families) that form pores in midgut cell membranes after their ingestion by dipteran larvae, leading to their death (Schnepf et al. 1998; de Maagd et al. 2001). The presence of several toxins acting in synergy is known to hinder resistance evolution in target species (Wirth et al. 2005).

In natural mosquito populations, no consistent resistance has been detected even after long-term treatment with *Bti* toxins (Goldman et al. 1986; Becker and Ludwig 1993), and only moderate *Bti* resistances were described locally (Zhang et al. 2004; Paul et al. 2005; Paris et al. 2010; Boyer et al. 2012). A laboratory-selected *Ae. aegypti* strain expressing resistance levels up to 30-fold for *Bti* Cry toxins was obtained from the susceptible Bora-Bora strain through repetitive generations of selection with field-collected leaf litter containing *Bti* toxins (Paris et al. 2011b). This strain offers the opportunity to investigate *Bti* toxin resistance mechanisms in this dengue vector species at genomic level (Bonin et al. 2009; Paris and Despres 2012). Furthermore, laboratory strains of the mosquitoes *Ae. aegyti* and *Culex pipiens* showed that high levels of resistance to *Bti* Cry toxins were associated with low resistance to the full *Bti* mixture (Georghiou and Wirth 1997; Paris et al. 2011b). This suggests that even a moderate resistance to the full *Bti* mixture measured in natural populations could hide much higher levels of resistance to some individual *Bti* Cry toxins. Although *Bti* resistance mechanisms in mosquitoes are still unknown, two major Cry toxin resistance mechanisms have been described in lepidopterans: a decrease in the activity of midgut proteases involved in toxin activation such as trypsins and modifications in membrane receptors of toxins such as cadherins, alkaline phosphatases, aminopeptidases and alpha-amylases (Gahan et al. 2001; Morin et al. 2003; Jurat-Fuentes and Adang 2004; Herrero et al. 2005; Bravo et al. 2007).

The rapid development of next-generation sequencing (NGS) technologies offers very promising applications for studying adaptation at the genomic scale, for both model and nonmodel species (Stapley et al. 2010). Thousands of single nucleotide polymorphisms (SNPs) can be discovered and analysed either by sequencing whole genomes (Turner et al. 2010; Fabian et al. 2012) or subsets of the genome using target sequencing (Albert et al. 2007; Nadeau et al. 2012), or random amplified DNA tags (Baird et al. 2008; Hohenlohe et al. 2010). Alternatively, transcriptome characterization with RNA sequencing can also be used to detect SNPs (Namroud et al. 2008; Renaut et al. 2010; Gagnaire et al. 2012). RNA sequencing is one of the most popular NGS applications in nonmodel species (Ekblom and Galindo 2011) and offers several advantages for the study of adaptation compared with genome sequencing: (i) this method focuses on coding regions that are the main targets for selection in the case of resistance linked to mutations in toxin receptors, (ii) it provides SNP data that can be easily transferred between populations and closely related species, (iii) both gene expression and gene sequence information are obtained, and (iv) it costs less than whole genome sequencing (Bonin 2008). Whilst NGS technologies are useful for generating large amounts of sequence data, the cost remains too high for most population genomics studies on nonmodel species. The pooled sample strategy offers the opportunity to obtain genomewide sequence data with high coverage in a short time span and low cost compared with individual sequencing. It is especially well suited to genome scan or genomewide association studies involving several populations and individuals (Van Tassell et al. 2008; Futschik and Schlotterer 2010; Ekblom and Galindo 2011). This approach has already shown high levels of reliability for DNA sequencing in humans and is now widely used for the screening of rare variants and disease-related allelic differences (Sham et al. 2002). This pooled approach has also been used successfully in many species including Arabidopsis (Turner et al. 2010), Drosophila (Kolaczkowski et al. 2011; Boitard et al. 2012; Fabian et al. 2012), fish (Renaut et al. 2010) and mosquitoes (Cheng et al. 2012) and has become one of the methods of choice for studying adaptation in natural populations.

In this study, we used pooled mRNA tag sequencing to detect genomewide SNPs in a susceptible and a *Bti*-resistant laboratory strain of *Ae. aegyti* in order to search for genomic regions and candidate genes exhibiting signatures of selection in the *Bti*-resistant strain. The frequencies of resistance alleles are expected to increase in the selected strain, resulting in a higher than expected genetic differentiation (*F*_st_) at these loci compared with the rest of the genome. Because of hitchhiking effects, positive selection for *Bti* resistance should lead to increase the differentiation not only in the genes involved in resistance but also in the flanking regions (Barton 2000). We validated the pooled strategy by individually genotyping 58 individuals for 372 detected SNPs using the DNA Illumina GoldenGate® array (Illumina Corporation, San Diego, CA, USA). We then sequenced three detected candidate genes, as well as four undetected candidate genes as controls, and looked for evidence of selection at sequence level.

Furthermore, we tested whether the discovery of hundreds of SNPs in the *Ae. aegyti* Bora-Bora laboratory strain could provide valuable tools for investigating *Bti* resistance mechanisms in natural populations of *Ae. aegyti* in Martinique (French West Indies) treated since 2007 with *Bti* (Vectobac®, water dispersible granules (WG), 3000 UTI/mg; Valent Biosciences, Libertyville, IL, USA). In Martinique, the dengue virus (Flaviviridae) transmitted by *Ae. aegypti* is a major public health issue (San Martin et al. 2010). With no vaccine or specific treatment for the disease, vector control using chemical or biological agents against *Ae. aegypti* is currently the main method of reducing dengue transmission. In this study, we first analysed the levels of resistance for four natural populations in Martinique treated with *Bti* since 2007 and two untreated populations. We then sequenced the 372 validated SNPs for these six populations from Martinique (30 individuals per population) and tested for association between genotypes and levels of *Bti* resistance. Finally, we compared the genomic regions linked to *Bti* resistance in the laboratory strain and in natural populations in Martinique, looking for common regions under selection in different genetic contexts.

## Materials and methods

### Laboratory mosquito strain selection

The *Ae. aegypti* laboratory strain Bora-Bora susceptible to all insecticides was selected using toxic leaf litter containing *Bti* toxins (LiTOX strain) as described by Paris et al. (2011b). For each generation, an average of 6000 larvae were exposed to toxic leaf litter in order to obtain a mortality rate of 70%. An average introgression rate of 2.5% of susceptible Bora-Bora mosquitoes at each generation was introduced to limit the effects of genetic drift in the selected strain. Mosquito strains were reared in standard insectarium conditions (27°C, 14 h/10 h light/dark period, 80% relative humidity). The average generation turnover was 45 days, and selection was carried out over 18 generations. After 10 generations of selection, the LiTOX strain experienced a sharp decrease in population size (Table S1). Selection was therefore interrupted for two generations, and an introgression of 20% of susceptible Bora-Bora mosquitoes was used for these two generations to limit bottleneck.

Larval bioassays revealed high resistance to all Cry toxins tested individually with a resistance ratio RR_50_ (i.e. the ratio between the 50% lethal concentration values at 24 h for the resistant and the susceptible strains) of 30.2-fold, 13.7-fold and 6.3-fold for Cry4A, Cry4B and Cry11A, respectively (Paris et al. 2011b). The LiTOX strain also showed moderate but significant resistance to the whole commercial *Bti* mixture with a RR_50_ of 2-fold.

### Sample preparation and mRNA sequencing

Three independent batches of 100 larvae from the Bora-Bora and the resistant LiTOX strain were reared in identical conditions as described above (three biological replicates). After 5 days (fourth stage larvae), 30 fresh larvae from each batch were used for total RNA extraction using the PicoPure™ RNA isolation kit (Molecular Devices, Sunnyvale, CA, USA) according to the manufacturer's instructions. Total RNA quality and quantity were measured using a NanoDrop ND-1000 (LabTech, East Sussex, UK) and an Agilent 2100 Bioanalyzer (Agilent Technologies, Santa Clara, CA, USA) before dilution to 750 ng/μL in nuclease-free water. For each strain, total RNA from the three biological replicates was then pooled together in equal proportions. One cDNA library for each strain was prepared from pools of total RNA as described by David et al. (2010) and by Paris et al. (2012). Two micrograms total RNA was used to isolate mRNAs using magnetic oligo(dT) beads before cDNA synthesis. Double-stranded cDNAs were cleaved at DpnII restriction sites, and gene expression adapters 1 and 2 were ligated to the DpnII cleavage and the MmeI cleavage site, respectively, using T4 DNA ligase. The adapter-ligated cDNA tag library was then enriched using PCR (30 s at 98°C followed by 15 cycles of 10 s at 98°C, 30 s at 60°C, 15 s at 72°C and a final elongation step of 10 min at 72°C) and gel-purified before quality control analysis on an Agilent 2100 Bioanalyzer. Each cDNA tag library was sequenced as 20-mers (mRNA tags) on a separated flow cell lane using a Genome Analyzer I (Illumina Corporation, San Diego, CA, USA).

### Read mapping, SNP calling and outlier detection in the laboratory strains

Sequenced cDNA tags were mapped on the *Ae. aegypti* genome assembly (AaegL 1.1 annotation) using tagmatcher, a software program based on the short sequence mapping algorithm ‘agrep’ (Wu and Manber 1992). tagmatcher makes it possible to match tags to a reference genome with errors and multiple matching loci (available on request from eric.coissac@inrialpes.fr). The mapping procedure only retained tags with no ambiguous nucleotides which mapped with a 0 or 1 mismatch at a unique genomic location (Paris et al. 2012). SNPs with a minimum read coverage of 10, present in at least two different reads (as recommended by Ingman and Gyllensten 2009) and showing a minimum allelic frequency of 1%, were called with the samtool mpileup program (Li et al. 2009) and kept for further analyses. *F*_st_ values were then estimated for each SNP using the PoPoolation2 program, which provides several tools for population genetic analyses of NGS pooled data (Futschik and Schlotterer 2010; Kofler et al. 2011). An empirical outlier approach was used to detect the most strongly differentiated SNPs (Akey et al. 2010; Kolaczkowski et al. 2011; Fabian et al. 2012). Firstly, the most strongly differentiated SNPs that fell into the 1% tail of the *F*_st_ distribution were detected as potential outliers as described by Akey et al. (2010) and Kolaczkowski et al. (2011). These outliers exhibited *F*_st_ values of > 0.66. Then, a two-sided Fisher's exact test was performed for all SNPs to estimate the significance of allele frequency difference between the resistant and susceptible strains. The main advantage of this test is that it accounts for sequencing coverage, as allele frequencies and *F*_st_ values may be influenced by sampling effects at low coverage. Furthermore, to avoid false positives due to the large number of Fisher's exact tests performed, the false discovery rate was calculated for all SNPs by adjusting *P*-values (*q*-values) using the ‘qvalue’ package in R (Dalmasso et al. 2005), as recommended by Fabian et al. (2012). Only SNPs falling into the 1% tail of the *F*_st_ distribution and showing *q*-values <0.005 were identified as outliers.

### SNP validation using DNA Illumina GoldenGate® array

Of the 400 SNPs showing the greatest difference in allelic frequency between the two laboratory strains, 372 were selected by Illumina on the basis of designability rank and%GC and included in a multiplexing DNA Illumina GoldenGate® array. Of these, 274 were located in predicted genes, and 98 in nongenic regions. All 11 SNPs previously detected as outliers regarding *Bti* resistance (see Results) were included in this array.

Total genomic DNA was extracted from 28 LiTOX larvae (from the same generation as larvae used for the cDNA tags) and from 30 Bora-Bora larvae (several generations after the generation used for cDNA tags) with the DNeasy tissue Kit (Qiagen GmbH, Hilden, Germany) according to the manufacturer's instructions. DNA concentration was determined for each sample using a NanoDrop™ ND-1000 and adjusted to 50 ng/μL. Genotyping was performed at the Genotyping Center GENTYANE in Clermont-Ferrand, France http://www.ibisa.net/plateformes/GENTYANE,101.html) according to the standard protocol for the DNA Illumina GoldenGate® array (Fan et al. 2006), and data were analysed using the software program Genome Studio® v.1.5.10 (Illumina Corporation). The clustering of genotypic classes was manually adjusted when necessary. To ensure the reliability of the genotyping data, we used GenCall50 (GC50), which indicates the reliability of each genotype call, GenTrain, which measures SNP cluster quality, and the call rate (CR), which represents the fraction of 58 samples successfully genotyped for a given SNP. We kept SNPs with GC50 higher than 0.35, GenTrain higher than 0.50 and CR higher than 0.50.

For SNPs with <10% missing data (283 SNPs), the concordance between allelic frequencies obtained by cDNA tag sequencing and SNP genotyping was tested for the LiTOX strain using a Pearson's correlation using the ‘stat’ package in the R software version 2.10 (R Development Core Team 2011). A Pearson's correlation was also performed between allelic frequencies considering only the SNPs detected as outliers for *Bti* resistance. Furthermore, a Fisher's exact test (with Bonferroni correction) was performed to identify significant differences between the allelic frequencies of the pooled and the individual data sets.

### Candidate gene identification, sequencing and neutrality tests

Candidate genes for *Bti* resistance were identified using a keyword search in the VectorBase database http://aaegypti.vectorbase.org/index.php. A total of 162 genes belonging to families of toxin receptors (14 alkaline phosphatases, 23 aminopeptidases, 17 cadherins and 20 alpha-amylases) and enzymes involved in toxin activation (six chymotrypsins and 82 trypsins) previously shown to be potentially involved in *Bti* resistance were considered as candidates. These candidate genes were dispersed over 94 supercontigs in the *Ae. aegypti* genome.

The three aminopeptidases (VectorBase Gene ID AAEL007892, AAEL004738 and AAEL008155) identified in a region showing a signature of selection were sequenced for at least nine susceptible and LiTOX individuals. We also sequenced as a negative control three candidate genes described in literature as binding Cry toxins (the aminopeptidase AAEL012778, the cadherin AAEL007488 and the alkaline phosphatase AAEL009077; Chen et al. 2009; Likitvivatanavong et al. 2011b) but located in genomic regions with low *F*_st_ between the susceptible and resistant strains, and the aminopeptidase AAEL004226 located more than 1 million bp from an outlier. The complete sequence for each candidate gene was downloaded from VectorBase, and primers amplifying about 1000 bp (range 669–1036) were designed in each gene using the software package Lasergene 7.2 (DNASTAR Inc., Madison, WI, USA) (Table S2). DNA amplification was performed in 25 μL total volume with 2 mm MgCl_2_, 0.1 mm of each dNTP (Roche Diagnostics, Basel, Switzerland), 0.2 μm of each primer, 5 μg BSA, 0.6 U AmpliTaq Gold DNA polymerase (Applied Biosystems, Foster City, CA, USA) and 50 ng DNA. The PCR program was initial 10 min denaturation step at 95°C; 40 cycles of denaturation at 95°C for 45 s, annealing at 58°C for 45 s and elongation at 72°C for 60 s; and a final extension step at 72°C for 5 min. Sequences performed by Genoscreen (http://www.genoscreen.fr) were aligned and corrected using the program Bioedit. Haplotype phase was inferred, and genetic diversity and differentiation analyses (nucleotide diversity *p*, haplotype diversity *H*_d_, number of segregating sites *S*, number of singletons *S*_i_, Watterson's mutation parameter θ_W_, average number of nucleotide differences *K* within and between strains) were performed using the software dnasp 5.0 (Librado and Rozas 2009). Deviation from neutral equilibrium expectations was tested by applying Tajima's D (Tajima 1989), Fu and Li's D* and F* (Fu and Li 1993) tests. To assess whether these statistics significantly departed from a neutral scenario of evolution given the resistant strain's known demographic history, we performed coalescent simulations using the ms program (Hudson 2002). This program generates random independent samples according to a Wright–Fisher neutral model allowing for population size changes at each generation. For each gene, the mutation rate μ was estimated from the per-locus mutation parameter observed for the susceptible strain (θ = 4*N*_e_μ and *N*_e_ = 6000) and used as the starting value for the simulations. Then, 1000 neutral samples consisting of 20–24 haplotypes, depending on the number of individuals sequenced in the LiTOX strain, were simulated based on the resistant strain's known demographic history (Bonin et al. 2009, Table S1).

### Natural populations from Martinique: sampling and bioassays

**Figure 1:**
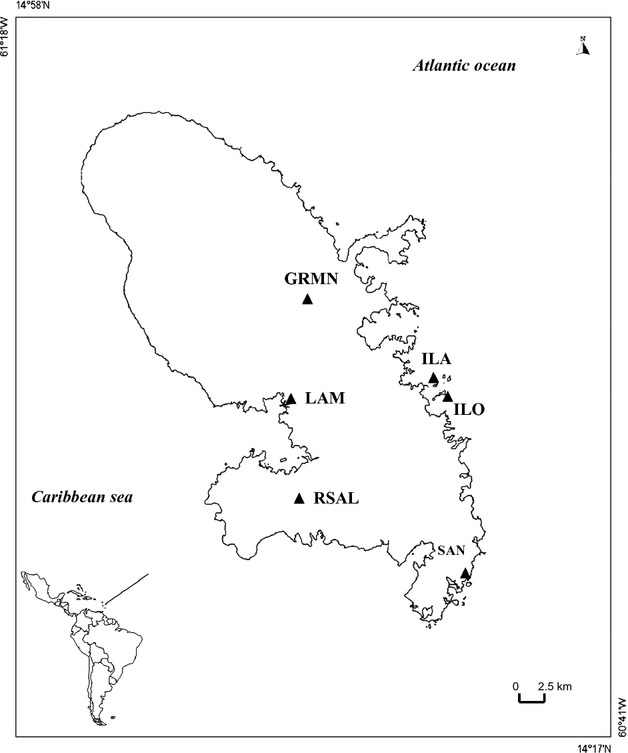
Geographical distribution of the six natural populations of *Aedes aegypti* sampled in Martinique Island. Four populations were collected on the main island, in the cities Gros Morne (GRMN), Lamentin (LAM), Rivière Salée (RSAL) and Saint Anne (SAN), and two on the small islands Ilet Long (ILO) and Ilet Anonyme (ILA) located 1 km from the Atlantic coast.

Larvae or pupae were collected from domestic breeding habitats in six natural populations of *Ae. aegypti* in the island of Martinique from January to March 2009. Four populations were located on the main island of Martinique in the cities Gros Morne, Lamentin, Rivière Salée and Saint Anne. These cities have been treated with *Bti* since 2007 with an average of around 30 treatments between 2007 and 2009. Furthermore, two untreated populations were collected on the small islands Ilet Long and Ilet Anonyme located at a distance of 1 km away from the Atlantic coast (Fig. 1). We collected both treated and untreated populations to maximize the range of variability in resistance levels between the populations. For each population, about one thousand larvae were reared in laboratory conditions until the adult stage and morphologically identified. The adults were kept in cages until mating, and the females were blood-fed on rabbits until they laid the large number of eggs needed for insecticide bioassays.

Bioassays were performed using the commercial *Bti* mixture Vectobac®12AS [1.2%, 1200 International Toxic Units (ITU) per milligram] on third-instar larvae following WHO protocols (WHO 2005) as described by Marcombe et al. (2012). Bioassays were replicated four times, and a minimum of five concentrations in the activity range of the insecticide were used (*n* = 521 in average per population). The bioassays were performed at two different dates: March 2009 for the populations Gros Morne, Lamentin, Rivière Salée and Saint Anne; and July 2009 for the populations Ilet Long and Ilet Anonyme. The susceptible Bora-Bora strain was included in both bioassays and used as a reference to determine the relative levels of resistance in the natural *Ae. aegypti* populations tested the same date (Bora-Bora 1 for March 2009, and Bora-Bora 2 for July 2009, see Table S3).

The lethal concentrations for 50% and 95% of individuals after 24 h' exposure (LC_50_ and LC_95_) and their 95% fiducial limits were calculated for each population using the log-probit method of Finney after correction with Abbott's formula implemented in the probit software (Raymond 1995). The resistance ratios RR_50_ (or RR_95_) were calculated by dividing the LC_50_ (or LC_95_) value obtained for each population with the LC_50_ (or LC_95_) value obtained for the Bora-Bora susceptible strain. The 95% confidence interval limits (95% CI) of RR_50_ (or RR_95_) were calculated by dividing the extreme 95% CI values of LC_50_ (or LC_95_) obtained for each population by those obtained for the Bora-Bora strain during the bioassays. For each population, resistance ratios to *Bti* were considered significantly different when the LC 95% fiducial limits did not overlap with those of the reference strain and the 95% CI of resistance ratios were higher than 1.

Furthermore, two linear mixed models (LMMs) were used to test whether the overall mortality was different in the Martinique populations compared with the susceptible Bora-Bora strain. The first LMM compared Bora-Bora 1 and the four Martinique treated populations, and the second one Bora-Bora2 and the two Martinique untreated populations. Analyses were performed with the R software version 2.10 (R Development Core Team 2011; function *lmer*, package *lme4*) as described by Boyer et al. (2012). For each replicate, larval mortality was determined as the number of dead larvae compared with the initial number of larvae used for the bioassays (25 larvae), and a quasibinomial error distribution was used for the LMMs analysis. Insecticide concentration during the bioassays was considered as a fixed factor, and the populations were considered as a random factor in the analyses. The accuracy of the LMMs was validated after the study of the QQ-plot of the observed residual distribution against the model distribution. Significances of LMMs were calculated using an analysis of variance using a likelihood ratio test (Fitzmaurice et al. 2004).

### Natural population genotyping and association mapping for *Bti* resistance

Total genomic DNA was extracted from 30 four-instar larvae per population with the DNeasy tissue Kit (Qiagen) and adjusted to 50 ng/μL before genotyping with the Illumina GoldenGate® array as described above. Association mapping analyses between *Bti* LC_95_ and SNPs were performed using the least squares fixed effects linear model in the software program tassel v3 (Bradbury et al. 2007). This model tests for associations between genotypes at each segregating marker and phenotype value, taking population structure into account. Population structure was estimated from the three principal components of a principal component analysis (PCA) based on a correlation matrix calculated from genetic markers, as recommended by Bradbury et al. (2007). The first three PCA axis and phenotype values were included in the linear model as fixed effects. *P*-values were corrected for multiple comparisons using permutation tests with 1000 permutations and a 0.05 level of confidence.

## Results

### SNP detection using mRNA tags

A total of 6.7 millions reads were sequenced across the two laboratory strains Bora-Bora and LiTOX, distributed over 6038 predicted genes and 4278 clusters outside the predicted gene boundaries (Paris et al. 2012). These regions detected outside gene boundaries (mentioned as nongenic regions in this article) may represent genes, exons or UTR extensions not predicted by automated annotation but also pseudogenes, transposable elements or noncoding RNAs with polyadenylated sequences (David et al. 2010). From a total of 1541 detected SNPs, 1520 were successfully selected using our criteria (see Methods): 1137 SNPs were located in 1078 predicted genes with a high mean coverage of 1179 reads per position across the two libraries, and 383 SNPs were located in nongenic regions with a mean coverage of 411 reads per position. The 1520 SNPs covered 580 supercontigs of the *Ae. aegypti* reference genome, which represents 36% of the 1612 supercontigs containing genes and 12% of the total number of 4758 supercontigs in the reference. These SNPs also covered 20 candidate genes for *Bti* resistance including 15 putative *Bti* toxin receptors and five enzymes potentially involved in toxin activation and/or degradation.

### SNP validation using DNA Illumina GoldenGate® array

DNA Illumina GoldenGate® arrays provided successful genotyping for 94.9% of the SNPs with an average of just 8.9% of missing data. Most of the SNPs detected by mRNA tag sequencing were also detected with the Illumina SNP array (96.3%): only 13 of 353 SNPs successfully genotyped were monomorphic in both strains while showing allelic frequencies ranging from 7 to 41% with mRNA tag sequencing. The Pearson's correlation between allelic frequencies obtained using both methods in the LiTOX strain was highly significant (*P* < 0.001, *r* = 0.85), validating the reliability of our mRNA tag sequencing pooled data for inferring population allelic frequencies. The correlation was even stronger when considering the allelic frequencies obtained for the SNPs detected as outliers (*P* < 0.001, *r* = 0.94). The Fisher's exact test showed that 99% of the SNPs showed no differences in allelic frequencies between the two methods, with only three SNPs showing significant differences in allelic frequencies (Figure S1).

The genotyping of natural populations from Martinique was successful for 324 of the SNPs (87.1%), with 48 SNPs discarded because of low quality profiles and a high proportion of missing data. A higher proportion of successful SNPs in natural populations was found when they were located in genes (88.4%) compared with nongenic regions (83.5%). The SNPs detected in the Bora-Bora laboratory strain were highly polymorphic in natural populations in Martinique with 95.9% of SNPs polymorphic in at least two individuals, and a mean allelic frequency of 14.3% across the six populations. Ten of the 13 SNPs found to be monomorphic in the laboratory strains were polymorphic in natural populations with frequencies ranging from 0.9 to 12.1%. Altogether, 99.2% of the SNPs detected by mRNA sequencing were validated either in the laboratory strains or in the natural populations.

### Outlier SNP and candidate gene detection in the laboratory strains

**Figure 2:**
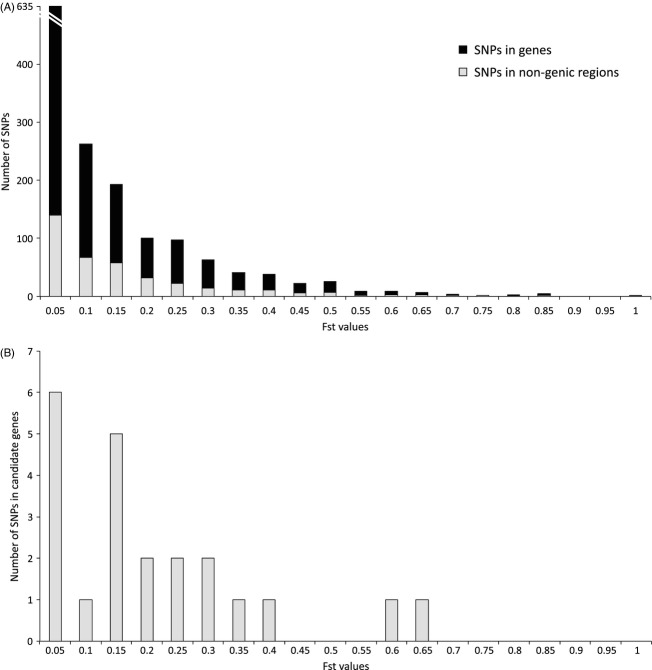
*F*st distribution of SNPs detected in the laboratory strains on genic and nongenic regions (A) and on candidate genes for *Bti* resistance (B).

**Figure 3:**
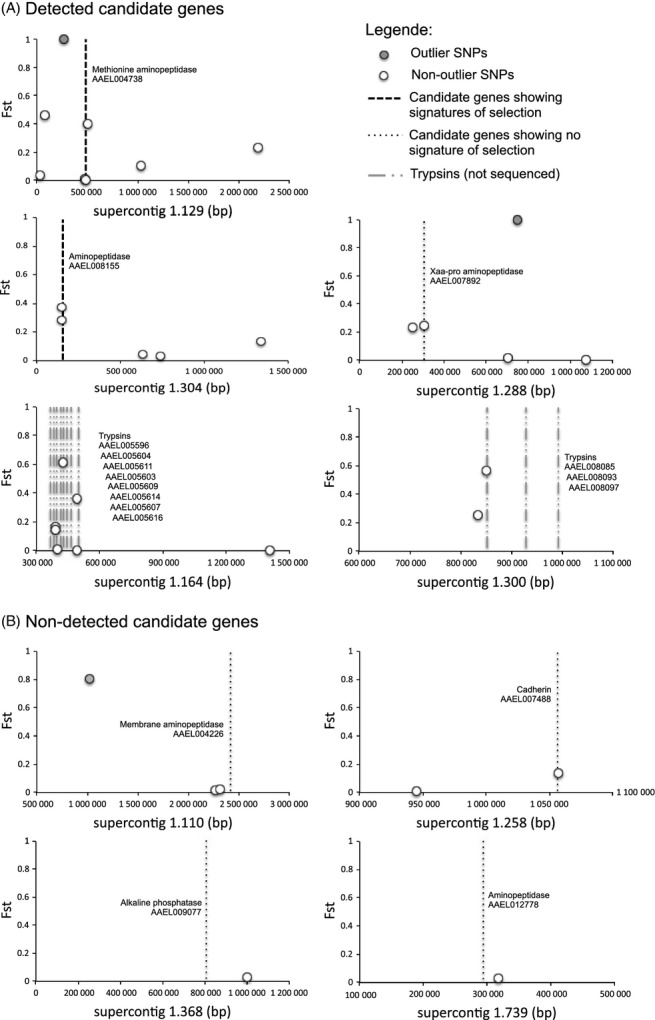
The genomic location of candidate genes detected on the LiTOX strain (A), and undetected candidate genes sequenced as negative controls (B).

*F*_st_ values between the susceptible and the resistant laboratory strains were highly variable across SNPs (Fig. 2), with average *F*_st_ values of 0.12 for SNPs located in predicted genes and 0.14 for SNPs located in nongenic regions. As similar *F*_st_ values were detected for genic and nongenic regions, no bias linked to faster evolutionary rates in nongenic regions is expected in the detection of outlier and SNPs located in both regions were kept for outlier analyses. Eleven of the 1520 loci had *F*_st_ in the 1% tail of the *F*_st_ distribution and significant difference in allelic frequencies (*q*-values < 0.005) between the two strains. These 11 outliers were located on different supercontigs of the *Ae. aegypti* genome (Table 1). Three were located in nongenic regions and eight in predicted genes: six in unknown proteins, one in a tRNA selenocysteine-associated protein and one in a fas-associated protein (Table 1). Three outliers were located in the same supercontigs as candidate receptors for *Bti* toxins (three aminopeptidases). The aminopeptidase AAEL004738 was located in the supercontig 1.129 at 219 442 bp from the outlier and at 11 338 bp and 409 981 bp from two SNPs with *F*_st_ > 0.4 in the same supercontig (Fig. 3). In the supercontig 1.288, the aminopeptidase AAEL007892 was located 443 317 bp from the outlier and itself contained a SNP with a significant difference in allelic frequency between the two strains (Fig. 3). Finally, the aminopeptidase AAEL004226 in the supercontig 1.110 was located far away from the outlier locus (1 392 068 bp), was located at 107 042 and 150 201 bp from 2 SNPs showing very low *F*_st_ values (0.019 and 0.026, respectively) and was therefore retained as a nondetected candidate for further analyses.

**Table 1:** Description and genomic location of the 11 SNPs detected as outlier in the laboratory strains. The results of the association mapping analyses performed on the six natural populations of the Martinique Island are also indicated

							Laboratory strains		Natural populations	
								
Marker name	Supercontig	Location (bp)	Alleles (protein change)	Gene no.	Gene function	Candidate genes in the supercontig	*F*st	*q*-value	*R*^2^	*P*-value
1.129_AAEL004770	1.129	268 132	A/G	AAEL004770	Hypothetical	Methionine aminopeptidase AAEL004738 (219 442 bp)	1	1.12E-03	0.037	1
1.288_AAEL007910	1.288	749 873	A/G	AAEL007910	Hypothetical	Xaa-pro aminopeptidase AAEL007892 (443 317 bp)	1	2.05E-05	0.129	0.001
1.70_AAEL002875	1.7	79 255	T/G	AAEL002875	Hypothetical	–	0.83	0.00E+00	0.04	1
1.88_AAEL003495	1.88	2 425 453	A/G	AAEL003495	Hypothetical	–	0.82	3.54E-05	0.006	1
1.278_NA0154	1.278	80 4286	T/C	Nongenic	–	–	0.82	2.83E-04	0.009	1
1.110_AAEL004220	1.11	1 026 779	A/G (Thr/Ala)	AAEL004220	Hypothetical	Membrane alanine aminopeptidase AAEL004226 (1 392 068 bp)	0.81	5.25E-07	0.1	0.038
1.146_NA0054	1.146	1 639 650	T/A	Nongenic	–	–	0.8	1.22E-15	0.005	1
1.113_AAEL004287	1.113	1 312 954	T/A	AAEL004287	fas-associated protein	–	0.79	1.27E-26	0.003	1
1.414_AAEL009633	1.414	1 016 051	T/C	AAEL009633	Hypothetical	–	0.78	1.51E-283	0.198	0.001
1.640_AAEL011988	1.64	43 654	A/G	AAEL011988	tRNA selenocysteine-associated protein	–	0.78	2.90E-07	0.053	0.793
1.39_NA0205	1.39	1 451 367	T/C	Nongenic	–	–	0.69	1.61E-16	7.13E-06	1

Single nucleotide polymorphisms were detected in 20 candidate genes for *Bti* resistance: one cadherin, three alkaline phosphatases, seven alpha-amylases, four aminopeptidases and five trypsins. On average, *F*_st_ values were higher in candidate genes than for the SNPs as a whole (*F*_st_ = 0.19) (Table S4). Three candidate genes contained SNPs with *F*_st_ values that were higher than 0.35 and significant allelic frequency difference between the two strains (*q*-value < 0.005): the aminopeptidase AAEL008155 (two SNPs, *F*_st_ = 0.38 and *F*_st_ = 0.28), and the two trypsins AAEL008097 (*F*_st_ = 0.56) and AAEL005609 (*F*_st_ = 0.62). The gene AAEL008155 is one of the *Ae. aegypti* aminopeptidases described in the literature as capable of binding the *Bti* toxin Cry11A and therefore represents a good candidate for *Bti* resistance in the LiTOX strain (Likitvivatanavong et al. 2011b). The two trypsins were located in supercontigs 1.164 and 1.300 each containing SNPs with *F*_st_ > 0.25 (Fig. 3). The supercontig 1.164 contained eight trypsins located from 0 to 67 000 bp from the outliers. It is expected that modifications in the level of expression rather than changes in the amino acid sequence of these enzymes are linked to *Bti* resistance. As we detected an outlier close to these genes, the regulatory elements might be located in this genomic region. However, as they are not known for these genes, it was not possible to sequence them.

### Candidate gene sequencing

The three detected candidate aminopeptidases exhibited levels of nucleotide diversity ranging from 0.0004 to 0.0165 in the susceptible strain (Table S5). A deletion of 5 bp leading to a premature breakdown of the protein synthesis was found in the gene AAEL007892 in both laboratory strains. For the gene AAEL008155, Fu and Li's D* and F* neutrality tests showed significant departure from neutrality for the LiTOX strain, even after correction according to coalescent simulations based on this strain's known demographic history (Table 2). Tajima's D-test was marginally significant for this gene (0.1 < *P*-value < 0.05). The positive values for Tajima's D, Fu and Li's D* and F* tests that reflect an excess of high frequency variants are compatible with ongoing positive selection in the LiTOX strain. A total of 32 and 30 segregating sites representing 35 mutations (eight nonsynonymous and 27 synonymous) and 31 mutations (seven nonsynonymous and 24 synonymous) were detected in Bora-Bora and LiTOX strains, respectively. The seven nonsynonymous mutations detected in the LiTOX strain were all present in similar proportions in the susceptible strain. Despite the low level of polymorphism (only three segregating sites, all synonymous), the gene AAEL004738 presented significant Fu and Li's D* and F* neutrality test results for both strains (Table 2), suggesting that the departure of neutrality in this gene is not linked to *Bti* resistance. None of the genes sequenced as negative controls showed consistent departure from neutrality (Table 2).

**Table 2:** Neutrality tests performed on the laboratory strains for three detected and four undetected candidate genes

	Susceptible strain		Resistant strain		
		
Neutrality test	Value	Significance	Value	Significance	Corrected significance
Detected candidate gene					
Xaa-pro aminopeptidase (AAEL007892)					
Tajima's *D*	0.45727	n.s.	1.1667	n.s.	
Fu and Li's D^^*^^	0.85471	n.s.	0.84065	n.s.	
Fu and Li's F^^*^^	0.87221	n.s.	1.08209	n.s.	
Methionine aminopeptidase (AAEL004738)					
Tajima's *D*	−1.73253	n.s.	−1.73253	n.s.	
Fu and Li's D^^*^^	−2.69255	*P* < 0.05	−2.69255	*P* < 0.05	*P* < 0.01
Fu and Li's F^^*^^	−2.83754	*P* < 0.05	−2.83754	*P* < 0.05	*P* < 0.01
Aminopeptidase (AAEL008155)					
Tajima's *D*	1.37086	n.s.	1.72156	n.s.	
Fu and Li's D^^*^^	0.92804	n.s.	1.7179	*P* < 0.02	*P* < 0.01
Fu and Li's F^^*^^	1.31688	n.s.	2.09343	*P* < 0.02	*P* < 0.01
Nondetected candidate sequences					
Membrane alanine aminopeptidase (AAEL004226)					
Tajima's *D*	1.00361	n.s.	1.85436	n.s.	
Fu and Li's D^^*^^	0.05455	n.s.	0.45445	n.s.	
Fu and Li's F^^*^^	−0.37147	n.s.	1.1342	n.s.	
Aminopeptidase (AAEL012778)					
Tajima's *D*	−0.45871	n.s.	−1.40392	n.s.	
Fu and Li's D^^*^^	1.08037	n.s.	1.63033	*P* < 0.05	*P* < 0.05
Fu and Li's F^^*^^	0.66979	n.s.	0.79391	n.s.	
Cadherin (AAEL007488)					
Tajima's *D*	0.13571	n.s.	−1.15933	n.s.	
Fu and Li's D^^*^^	1.09931	n.s.	−1.65357	n.s.	
Fu and Li's F^^*^^	0.95261	n.s.	−1.76132	n.s.	
Alkaline phosphatase (AAEL009077)					
Tajima's *D*	0.45727	n.s.	–	–	n.s.
Fu and Li's D^^*^^	0.85471	n.s.	–	–	n.s.
Fu and Li's F^^*^^	0.87221	n.s.	–	–	n.s.

Significance, significance according to coalescent simulations based on a large constant population size; corrected significance, corrected significance according to coalescent simulations based on the known demographic history of the resistant LiTOX strain; –, no polymorphism in the LiTOX strain.

### Association with *Bti* resistance in natural populations in Martinique

Bioassays showed a significant resistance ratio for all the natural populations treated with *Bti* compared with the reference susceptible Bora-Bora strain (no overlap of the LC_50_ and LC_95_ 95% fiducial limits, and 95% CI of RR_50_ and RR_95_ were > 1). This result was confirmed by the LMM analyses that detected a significant difference in mortality of the treated populations compared with the Bora-Bora strain (*t* value = 107.1, *P*-value < 0.001). These four populations showed varying levels of sensitivity to *Bti*, ranging from the RSAL population with a low but significant RR_50_ of 1.4-fold (LMM analyses: *t* value = 60.9, *P*-value < 0.001) to the SAN population with a RR_50_ close to the laboratory-selected LiTOX strain's RR_50_ (2.2-fold and 2-fold, respectively, Table 3 and Table S3). The two untreated ILO and ILA populations were susceptible to *Bti* and showed similar LC_50_ as the susceptible reference Bora-Bora strain. Furthermore, LMM analyses detected no significant difference between the mortality of these two populations and the reference Bora-Bora (*t* value = −14.8, *P*-value = 0.217).

**Table 3:** Description and resistant ratio (RR_50_) of the six natural populations of the Martinique Island and the selected laboratory strain LiTOX

Identity	Name	Code	GPS coordinates	*Bti* treatment status	RR_50_*	95% CI of RR_50_
Natural populations	Rivière Salée	RSAL	−60°59′3″/14°30′32″	Treated	**1.40**	1.23–1.62
	Lamentin	LAM	−60°59′33″/14°37′0″	Treated	**1.88**	1.52–2.36
	Gros Morne	GRMN	−60°59′20″/14°41′51″	Treated	**1.88**	1.64–2.20
	St Anne	SAN	−60°50′7″/14°26′39″	Treated	**2.27**	1.99–2.66
	Ilet Anonyme	ILA	−60°51′40″/14°37′8″	Untreated	1.03	0.88–1.18
	Ilet Long	ILO	−60°5′14″/14°36′42″	Untreated	0.85	0.74–0.97
Laboratory-selected strain†	LiTOX	LiTOX	–	Selected with field-collected leaf litter containing *Bti* toxins	**2.00**	1.80–2.30

* Significant resistance ratios are represented in bold.

† Data published in Paris et al. (2011b).

Association tests performed in the natural populations in Martinique detected 14 SNPs (4.3%) associated with *Bti* resistance. They were distributed across 14 supercontigs of the *Ae. aegypti* reference genome and located in 10 genes and four nongenic regions (Table 4). One of the SNPs associated with *Bti* resistance was located in a putative receptor for *Bti* Cry toxins, the alkaline phosphatase AAEL003298. This SNP is nonsynonymous, inducing a change from a threonine to a serine in position 496 of the alkaline phosphatase. A total of eight alkaline phosphatase genes were located up to 27 007 bp from this locus. Three of the 14 SNPs associated with *Bti* resistance in Martinique were detected as outliers in the laboratory-selected strain (Table 4).

**Table 4:** Description and genomic location of the 14 SNPs associated with *Bti* resistance in the natural populations in Martinique. Their *F*st values between laboratory strains are also indicated

Marker name	Supercontig	Location (bp)	Alleles (protein change)	Gene no.	Gene function	Candidate genes in the supercontig	*R*^2^	*P*-value	Laboratory strains *F*_st_
1.414_AAEL009633	1.414	1 016 060	T/C	AAEL009633	Hypothetical		0.20	0.001	0.78*
1.83_AAEL003298	1.83	1 254 086	T/A (Ser/Thr)	AAEL003298	Alkaline phosphatase	8 alkaline phosphatases	0.19	0.001	0.19
1.1168_AAEL014562	1.1168	182 816	T/C	AAEL014562	60S ribosomal protein L12		0.19	0.001	0.39
1.1002_AAEL014080	1.1002	176 807	A/G	AAEL014080	Aldehyde dehydrogenase		0.18	0.001	0.18
1.541_AAEL011089	1.541	250 285	G/C	AAEL011089	Ribonucleoprotein		0.16	0.001	0.27
1.68_NA0308	1.68	1 416 502	A/C	NA0308	–		0.14	0.001	0.10
1.288_AAEL007910	1.288	749 873	A/G	NA0158	Hypothetical	Xaa-pro aminopeptidase	0.13	0.001	1.00*
1.453_AAEL010112	1.453	564 695	A/G	AAEL010112	Hypothetical		0.11	0.001	0.17
1.4_AAEL000245	1.4	5 094 449	T/C	AAEL000245	Hypothetical	1 cadherin, 1 trypsin	0.10	0.001	0.45
1.274_NA0153	1.274	938 912	A/T	NA0153	–		0.11	0.002	0.38
1.46_AAEL001930	1.46	266 697	A/G	AAEL001930	pra1 protein	4 trypsin	0.09	0.02	0.19
1.625_NA0292	1.625	516 264	A/C	NA0292	–		0.08	0.033	0.44
1.110_AAEL004220	1.110	1 026 779	A/G (Ala/Thr)	AAEL004220	Hypothetical	Membrane alanine aminopeptidase	0.10	0.038	0.81*
1.177_NA0087	1.177	1 027 242	T/A	NA0087	–		0.09	0.041	0.30

* SNPs also detected as outliers in the laboratory-resistant strain.

## Discussion

### Using mRNA tags from pooled individuals to detect selection events

Using a pooled mRNA tag sequencing approach, we obtained 1520 SNPs sequenced with high coverage (986 on average) and distributed over 580 supercontigs of the *Ae. aegypti* genome. A total of 1078 genes, including 20 candidate genes for *Bti* resistance, and 383 nongenic regions were covered by this approach.

The very high reliability of the pooled sample approach for DNA sequencing in humans has already been demonstrated with Pearson's correlation coefficients up to 0.999 between expected and obtained allelic frequencies (Out et al. 2009; Bansal et al. 2011). Our results show that the use of the pooled sample strategy is also accurate when using mRNA sequencing of individuals reared in standardized laboratory conditions to detect genomewide polymorphism and to estimate allelic frequencies in nonmodel species. In this study, 96.3% of the SNPs detected using pooled mRNA sequencing were validated in the laboratory strains, and 99.2% either in the laboratory strains or in the natural populations. The proportion of validated SNPs was high compared with previous studies on nonmodel species in which 58–89.2% of SNPs were detected using mRNA sequencing (Wang et al. 2008; Canovas et al. 2010; Hubert et al. 2010; Milano et al. 2011). The similarity between our mRNA and DNA sequences is concordant with previous results in humans showing that RNA editing is a rare process and that very few differences between RNA and DNA can be found in the genome (Schrider et al. 2011).

A Pearson's correlation coefficient *r* = 0.85 was found between pooled mRNA and individual genotyping allelic frequencies, with only three SNPs showing significant differences between the two methods. This result is comparable with values obtained in human osteoblasts (*r* = 0.77) comparing allele difference estimations between DNA and mRNA pools (Verlaan et al. 2009). Biases in allelic frequency estimations using mRNA pools sequencing can be expected because a fraction of genes can experience alternative splicing (Graveley 2001; Trapnell et al. 2009) or random monoallelic expression (Gimelbrant et al. 2007). Furthermore, differential gene expression between samples is expected to affect the accuracy of allelic frequency estimations, especially when the samples are collected directly in the field or if they experiment different environments before RNA extraction.

### Outlier and candidate gene detection in the laboratory strains

Mean *F*_st_ values of 0.12 and 0.14 were obtained between the two laboratory strains for SNPs detected in genes and in nongenic regions, respectively. These results are concordant with a previous genome scan performed on the same two strains using 432 AFLP markers (*F*_st_ = 0.11, Paris and Despres 2012). Eleven outliers showed higher than expected genetic differentiation between the two strains, representing only 0.72% of the 1520 detected SNPs. This proportion was lower than a previous AFLP genome scan performed on the same strains (3.2%, Paris and Despres 2012), suggesting that the outlier selection in the present study was much more conservative and that we have probably missed some SNPs linked to *Bti* resistance (‘false negatives’).

The detected outliers were located in 11 supercontigs of *Ae. aegypti* genome, including two supercontigs already detected as being linked to *Bti* resistance in the same strains using polymorphic markers anchored on a Pony transposable element (supercontigs 1.414 and 1.288, Bonin et al. 2009). Although none of the outliers showing very high *F*_st_ between the resistant and susceptible strains were located in a candidate gene, two were physically close to two aminopeptidases (AAEL007892 and AAEL004738). Furthermore, three candidate genes of the 20 containing SNPs showed significant *F*_st_ > 0.35, including one aminopeptidase (AAEL008155) already described in the literature as binding Cry11A toxins (Likitvivatanavong et al. 2011b). Aminopeptidases are localized in the brush border of midgut epithelial and catalyse the cleavage of amino acids from the N-terminus of proteins. They are mainly involved in the digestion and transport of proteins ingested by larvae (Taylor 1993; Terra and Ferreira 1994). Regarding *Bti* resistance, they may play a role in protoxin activation and are considered as one of the putative *Bti* Cry toxin receptors, notably aminopeptidase N that has been described as a membrane binding protein for several Cry toxins in lepidopterans (Pigott and Ellar 2007) and mosquito species (Abdullah et al. 2006; Fernandez et al. 2006; Zhang et al. 2008; Likitvivatanavong et al. 2011a). In this study, the sequence of the two genes AAEL007892 and AAEL004738 did not show evidence of positive selection linked to *Bti* resistance, suggesting that other genes in the detected supercontigs might be the actual targets of selection. Neutrality tests performed on the aminopeptidase AAEL008155 showed significant departure from neutrality in the LiTOX strain but not in the susceptible strain. This significant departure from neutrality was observed even after correction according to coalescent simulations based on the known demographic history of the resistant LiTOX strain. This suggests that it is linked to the positive selection caused by *Bti* rather than to demographic effects. Furthermore, the analyses of the genes sequenced as a negative control confirmed that the positive selection signal is not detected over the whole genome as would be expected for demographic effects. A total of seven nonsynonymous mutations were detected in the sequence of the gene AAEL008155. None of them were fixed or significantly different in frequency in the resistant strain. This is probably because the resistance is not fixed after just 18 generations of selection, as shown by toxicological assays (Paris et al. 2011a). This gene represents a promising candidate for *Bti* resistance in the laboratory strain, and further analyses of the effect of the nonsynonymous changes in the entire protein structure and function would help to better understand its putative role in *Bti* resistance. However, this gene was not associated with *Bti* resistance in natural populations (*R*^2^ = 0.02; *P*-value = 1), suggesting that it is not involved in *Bti* resistance in Martinique.

Two genes encoding trypsins contained SNPs with significant *F*_st_ > 0.35. One was located in the supercontig 1.164 at <67 000 bp from seven other trypsins, including the gene AAEL005603 shown to be 14-fold overexpressed in the LiTOX strain (Paris et al. 2012). Several serine proteases such as trypsins and chymotrypsins present in insect midgut are involved in the activation and/or degradation of Cry toxins in lepidopterans (Pang and Gringorten 1998; Bah et al. 2004). The finding of high genetic differentiation associated with gene expression differences between the two strains for some of these trypsins suggests that modifications in the processing of protoxins could contribute to Cry toxin resistance in the LiTOX strain.

### *Bti* resistance in natural populations

Natural populations from Martinique treated with *Bti* for 2 years showed variable levels of resistance ranging from 1.4-fold to 2.26-fold, with populations exhibiting similar level of resistance than that observed in the selected laboratory LiTOX strain. Such levels of resistance obtained after <40 generations of *Bti* use in Martinique suggest that resistance was selected from standing genetic variation in these populations. The untreated populations located on small islands one kilometre from the Atlantic coast were not resistant to *Bti*, suggesting that low gene flow from treated populations and/or resistance costs linked to *Bti* resistance (Paris et al. 2011a) limit resistance evolution in these populations.

Single nucleotide polymorphism transferability from the Bora-Bora laboratory strain to the natural populations in Martinique was very high with more than 87% of the SNPs successfully genotyped and 96% of these showing polymorphism. This demonstrates the efficiency of the strategy used for SNP genotyping in natural populations of *Ae. aegypti*. Transferability was lower for SNPs located in nongenic regions, probably due to higher polymorphism in these regions meaning priming events were missed for the primers used for the DNA Illumina GoldenGate® array.

A total of 14 SNPs (4.3%) associated with *Bti* resistance were detected by association tests in the natural populations. One of them was located in the alkaline phosphatase AAEL003298 and induced a change from a threonine to a serine in populations showing higher levels of resistance. This type of modification in the amino acid sequence might modify the alkaline phosphatase affinity for one of the *Bti* toxins, and further functional analyses are needed to confirm this mutation's role as regards resistance. Indeed, eight other alkaline phosphatases, including one capable of binding Cry11B (gene AAEL003286, Likitvivatanavong et al. 2011b), are found very close to this SNP and may also be involved in resistance. Physical aggregation of candidate genes hinders the identification of the specific genes involved in resistance but more detailed sequencing of this entire region, as well as functional analyses on these nine genes, could help to go further in identifying the gene(s) and the mutation(s) involved in *Bti* resistance.

### Common genetic regions detected in laboratory strains and natural populations

Only three of the 11 outlier loci significantly differentiated between the laboratory strains were correlated with *Bti* resistance in the Martinique natural populations. This result suggests that few common genetic regions were selected from standing variation in two independent genetic contexts and that most of the genes associated with resistance might differ between the two environments. This only partial overlap between the outliers found in the laboratory strain and the SNPs associated with resistance in natural populations is not unexpected. Resistance to *Bti* in the LiTOX strain was shown to be multigenic (Paris et al. 2011b), in concordance with the selective pressure exerted simultaneously by several toxins and the high number of potential resistance mechanisms for each of them (Griffitts and Aroian 2005; Bravo et al. 2007). Therefore, resistance to *Bti* is expected to be controlled by a large number of genes with a small effect and may involve multiple but not necessarily substantial allele frequency changes (Le Corre and Kremer 2012). In such context, different genes can be selected in different environments and genetic backgrounds, and *F*_st_-based detection methods are likely to be poor to identify these genes under selection.

In contrast, the finding of three genomic regions identified as under selection in two independent genetic contexts recently affected by *Bti* selection pressure is a strong indication that they contain key genes for *Bti* resistance. Two of the three common SNPs are located in genomic regions previously detected as outliers in the LiTOX strain using polymorphic markers anchored on a Pony transposable element (Bonin et al. 2009). The detection of common genomic regions and genes linked to *Bti* resistance using different approaches and/or independent mosquito lineages (the Bora-Bora laboratory strain originates from Polynesia) suggests the consistency of genome scan approaches in detecting selection events on these regions. One of the repeatedly detected genomic regions is supercontig 1.288 containing nine genes with unknown functions and one putative candidate gene for *Bti* resistance, the aminopeptidase AAEL007892 sequenced in this study. However, the low sequence variability of this gene, along with the presence in both laboratory strains of a 5-bp deletion leading to a premature breakdown of the protein synthesis, suggests that this gene is probably not involved in *Bti* resistance in the laboratory strain. The second SNP common to both approaches is located in the gene AEL009633 annotated as an unknown protein with an orthologous gene in the mosquito species *Culex quinquefasciatus* coding for a pre-mRNA splicing factor (gene CPIJ014827). This gene is significantly over-transcribed (7.6-fold) in the LiTOX strain (Paris et al. 2012), suggesting that changes in mRNA splicing could contribute to *Bti* resistance. Indeed, alternative mRNA splicing could offer a fast adaptive response to strong selective pressure encountered by natural populations in the field, such as pesticides.

## Conclusion

This study demonstrates the suitability and accuracy of SNPs derived from mRNA tag sequencing to perform a genome scan of thousands of genetic markers distributed all along the genome. Using a comparison between a laboratory susceptible strain and a strain resistant to the bioinsecticide *Bti* followed by candidate gene sequencing, we detected the aminopeptidase AAEL008155 with significant departure from neutrality at the sequence level in the resistant strain. This aminopeptidase was already described in the literature as binding *Bti* Cry11A toxins and represents a good candidate gene for *Bti* resistance in the laboratory strain. Furthermore, this study shows that the SNPs detected in the laboratory strains using mRNA tag sequencing can be quickly converted in a SNP array with very high transferability to natural *Ae. aegypti* populations. A SNP-based association mapping on six populations from Martinique detected 14 loci associated with *Bti* resistance. Only three of them were detected as outliers in the laboratory-selected strain. Although the low overlap suggests that most of genes selected in mosquitoes with different genetic backgrounds differ, it also suggests that common selected regions might harbour key genes for *Bti* resistance.

## Data archiving statement

Next-generation sequencing data and cDNA library information associated with the present study have been deposited at the EMBL-EBI European Read Archive (ERA) under accession number ERA000115 and are freely accessible at http://ftp.era-xml.ebi.ac.uk/ERA000. Sequence data for this study are available in Dryad: doi: 10.5061/dryad.6s5pn.

## Supporting Information

**Figure S1.** Fisher's exact test values for the comparisons between allelic frequencies obtained using mRNA tag sequencing of pooled data and individuals DNA Illumina GoldenGate® array genotyping.).

**Table S1.** Demographic history of the *Aedes aegypti Bti*-resistant strain.

**Table S2.** Sequences of primers used for candidate gene sequencing.

**Table S3.** Resistance levels of the six natural populations from Martinique Island.

**Table S4.** Description and differentiation values between the laboratory strains of SNPs detected in candidate genes.

**Table S5.** DNA variation and divergence at sequenced loci for the laboratory strains.
