# Supplementary material for: Investigating the genetics of Bti resistance using mRNA tag sequencing: application on laboratory strains and natural populations of the dengue vector Aedes aegypti
Source: Evol Appl. 2013 Aug 31;6(7):1012–27. doi: 10.1111/eva.12082 (PMC3804235; doi:10.1111/eva.12082)
Supplement: Supplementary file 1 [file eva0006-1012-SD1.docx]

## Table S1: Demographic history of the *Aedes aegypti* *Bti*-resistant strain LiTOX.

| **Generation** | **Selection** | **Population effective size** |
| --- | --- | --- |
| 0 (Susceptible strain) | Yes | 6000 |
| 1 | Yes | 300 |
| 2 | Yes | 300 |
| 3 | Yes | 300 |
| 4 | Yes | 300 |
| 5 | Yes | 300 |
| 6 | Yes | 300 |
| 7 | Yes | 300 |
| 8 | Yes | 300 |
| 9 | Yes | 300 |
| 10 | No* | 25 |
| 11 | No* | 1000 |
| 12 | Yes | 1000 |
| 13 | Yes | 1000 |
| 14 | Yes | 1000 |
| 15 | Yes | 1000 |
| 16 | Yes | 1000 |
| 17 | Yes | 1000 |
| 18 | Yes | 1000 |
| 19 | Yes | 1000 |
| 20 (Resistant strain) | - | 2000 |

* The resistant strain experienced a strong decrease in population size after 10 generations of selection, so selection was interrupted for two generations.
