# Supplementary material for: Investigating the genetics of Bti resistance using mRNA tag sequencing: application on laboratory strains and natural populations of the dengue vector Aedes aegypti
Source: Evol Appl. 2013 Aug 31;6(7):1012–27. doi: 10.1111/eva.12082 (PMC3804235; doi:10.1111/eva.12082)
Supplement: Supplementary file 2 [file eva0006-1012-SD2.docx]

**Table S2:** Sequences of primers used for candidate gene sequencing.

| **Genes** | **Product lenght** | **Primer names** | **Sequence (5'-3')** |
| --- | --- | --- | --- |
| Aminopeptidases |  |  |  |
| AAEL007892 | 991 | AAEL007892_exon4_F | GCCGAGCAACGAATCCAACTGT |
|  |  | AAEL007892_exon4_R | CCCTTTTCTCCATCCCATTTATTT |
| AAEL004738 | 669 | AAEL004738_exon3_F | ACGAAACAATTACAAACGCATACT |
|  |  | AAEL004738_exon3_R | ATCGGAGCCGCCATTCAGGA |
| AAEL004226 | 1031 | AAEL004226_exon1_F | CGGCGAGCACTTCATAACCAATA |
|  |  | AAEL004226_exon1_R | GAGCCCGGAGTCAGGAATAGTG |
| AAEL012778 | 916 | AAEL012778_exon1_F | TGCGGCAAAATCGGGAATGG |
|  |  | AAEL012778_exon1_F | GTACGGCACGGTGGCAGAACG |
| AAEL008155 | 844 | AAEL008155_exon6_F | GGAAACCGCCTACCCACCAT |
|  |  | AAEL008155_exon6_R | ACGGCCACTGTTTCACTGTTTGT |
| Cadherin |  |  |  |
| AAEL007488 | 760 | AAEL007488_exon5-7_F | CGGCCTCGTCGTTGCTAATGA |
|  |  | AAEL007488_exon5-7_R | ATCGCCACCAATAACGCTAACG |
| Alkaline phosphatase |  |  |  |
| AAEL009077 | 1036 | AAEL009077_exon3_F | CTCGGCGACGGCTTATTTGTGC |
|  |  | AAEL009077_exon3_R | GTCTCCGCCGTGGGTTTCGTA |
