# Supplementary material for: Investigating the genetics of Bti resistance using mRNA tag sequencing: application on laboratory strains and natural populations of the dengue vector Aedes aegypti
Source: Evol Appl. 2013 Aug 31;6(7):1012–27. doi: 10.1111/eva.12082 (PMC3804235; doi:10.1111/eva.12082)
Supplement: Supplementary file 3 [file eva0006-1012-SD3.docx]

**Table S4:** Description and differentiation values between the laboratory strains of SNPs detected in candidate genes.

| **Marker name** | **Gene** | **Supercontig** | **Gene function** | ***F*st** | **q-value** |
| --- | --- | --- | --- | --- | --- |
| 1.164_AAEL005609 | AAEL005609 | 1.164 | trypsin | 0.61516204 | 0.001802 |
| 1.300_AAEL008097 | AAEL008097 | 1.300 | trypsin-eta, putative | 0.56276022 | 7.73E-26 |
| 1.304_AAEL008155_B | AAEL008155 | 1.304 | aminopeptidase | 0.37662338 | 0.001733 |
| 1.13_AAEL000642 | AAEL000642 | 1.13 | alpha-amylase | 0.33655112 | 0.073787 |
| 1.304_AAEL008155_A | AAEL008155 | 1.304 | aminopeptidase | 0.28239466 | 0.075502 |
| 1.485_AAEL010537 | AAEL010537 | 1.485 | alpha-amylase | 0.26885388 | 8.41E-132 |
| 1.1232_AAEL014710 | AAEL014710 | 1.1232 | alpha-amylase | 0.24966829 | 0.379975 |
| 1.288_AAEL007892 | AAEL007892 | 1.288 | xaa-pro aminopeptidase | 0.24741508 | 0.001409 |
| 1.83_AAEL003298 | AAEL003298 | 1.83 | alkaline phosphatase | 0.18797816 | 2.09E-08 |
| 1.164_AAEL005611_A | AAEL005611 | 1.164 | trypsin | 0.16741788 | 2.12E-31 |
| 1.164_AAEL005611_B | AAEL005611 | 1.164 | trypsin | 0.14599686 | 0.586352 |
| 1.258_AAEL007488 | AAEL007488 | 1.258 | cadherin | 0.13922255 | 1.90E-04 |
| 1.273_AAEL007673 | AAEL007673 | 1.273 | alpha-amylase | 0.13628453 | 4.82E-27 |
| 1.100_AAEL003905 | AAEL003905 | 1.100 | alkaline phosphatase | 0.13297488 | 0.054555 |
| 1.175_AAEL005808 | AAEL005808 | 1.175 | alanyl aminopeptidase | 0.10039506 | 8.92E-04 |
| 1.326_AAEL008456 | AAEL008456 | 1.326 | alpha-amylase | 0.09575429 | 8.37E-01 |
| 1.739_AAEL012783 | AAEL012783 | 1.739 | aminopeptidase | 0.02975420 | 6.14E-09 |
| 1.485_AAEL010540 | AAEL010540 | 1.485 | alpha-amylase | 0.01910828 | 0.001604 |
| 1.306_AAEL008214 | AAEL008214 | 1.306 | trypsin, putative | 0.01772013 | 7.17E-04 |
| 1.83_AAEL003309 | AAEL003309 | 1.83 | alkaline phosphatase | 0.00730941 | 0.536884 |
| 1.164_AAEL005616 | AAEL005616 | 1.164 | trypsin | 0.00426616 | 0.034944 |
| 1.326_AAEL008451 | AAEL008451 | 1.326 | alpha-amylase | 0.00099638 | 0.006108 |
