# Supplementary figures and images for: Investigating the genetics of Bti resistance using mRNA tag sequencing: application on laboratory strains and natural populations of the dengue vector Aedes aegypti
Source: Evol Appl. 2013 Aug 31;6(7):1012–27. doi: 10.1111/eva.12082 (PMC3804235; doi:10.1111/eva.12082)

No. of comparisons

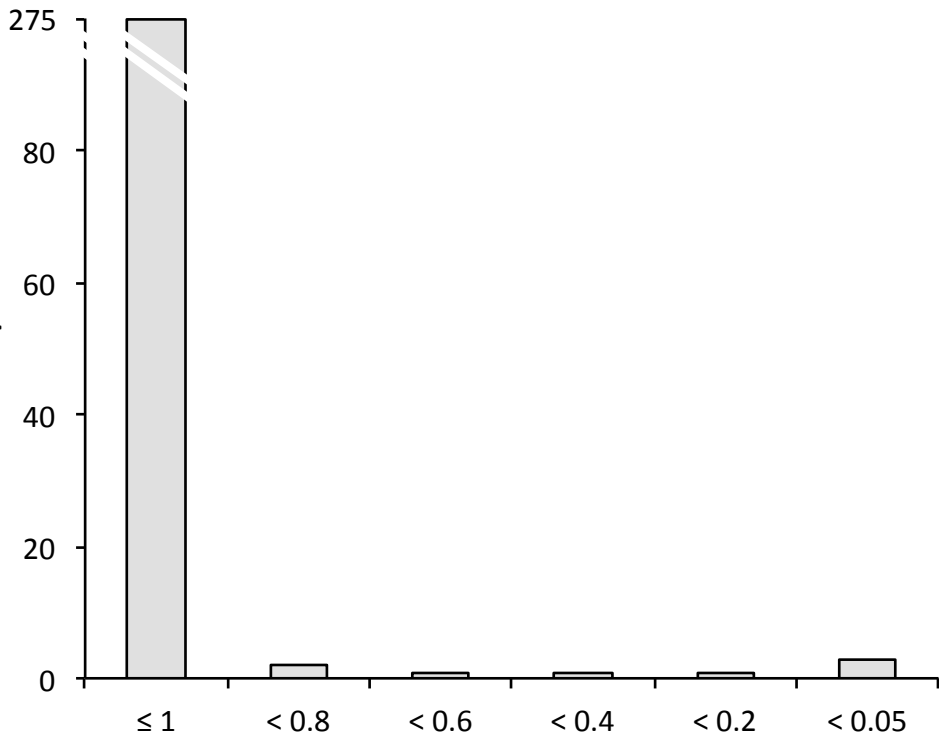

Fisher's Exact Test  $P$  values

Supplement: Supplementary file 4 [file eva0006-1012-SD4.pdf]
